# Untangling rapunzel syndrome: A unique presentation of gastric trichobezoar

**DOI:** 10.1016/j.ijscr.2024.110714

**Published:** 2024-12-16

**Authors:** Krati Agarwal, Radhika Agarwal, Vinayak Agarwal, Sonal Ratnakar Goel

**Affiliations:** a Department of Microbiology, AIIMS, Gorakhpur, India; b Department of Pathology, M.S. Pathology, Gorakhpur, India; c Department of surgery, Munshiratan cancer and G.I. clinic, Gorakhpur, India; d Department of Pathology, Health Plus Diagnostics, Gorakhpur, India

**Keywords:** Case report, Rapunzel syndrome, Trichobezoar, Adolescence

## Abstract

**Introduction and importance:**

Rapunzel syndrome is a rare condition that results from trichotillomania (compulsive hair pulling) and trichophagia (hair eating), causing a trichobezoar (hairball) to form This syndrome typically affects young females with psychiatric conditions and presents with symptoms like chronic abdominal pain, nausea, vomiting, and malnutrition. The condition is often diagnosed late, leading to serious gastrointestinal complications.

**Case presentation:**

A 19-year-old female from a rural community presented with chronic abdominal pain, vomiting, and nutritional deficiencies, including scaly skin and koilonychia. Over time, her symptoms worsened, and she discovered a palpable abdominal mass. Clinical evaluation, including an upper gastrointestinal endoscopy, revealed a large trichobezoar extending from the lower esophagus to the pylorus. The patient had a history of pica and compulsive behaviors, suggesting psychiatric involvement.

**Clinical discussion:**

The endoscopy revealed a 20 × 13.5 × 9 cm trichobezoar. After successful surgical removal, the patient's gastrointestinal symptoms improved. Post-operatively, she received nutritional support and was referred for psychiatric evaluation to manage trichotillomania and trichophagia, with the aim of preventing recurrence in a private practice setting.

**Conclusion:**

This case highlights the need for early recognition of Rapunzel syndrome in patients with chronic gastrointestinal symptoms and nutritional deficiencies. A multidisciplinary approach is essential for effective management and preventing recurrence.

## 1 Introduction

Bezoars are masses that form from indigestible food or foreign substances in the gastrointestinal tract. These masses can arise from leafy vegetables (phytobezoar), medications like antacids (pharmacobezoar), or substances such as hair and fat (trichobezoar). Bezoars are most commonly found in the stomach, particularly in patients who have undergone gastric surgery. In cases where there is no history of surgery, the underlying cause is often a psychiatric disorder like trichotillomania [1].

Trichobezoars are more commonly found in children and adolescents with normal gastrointestinal function and usually result from an underlying behavioral disorder known as “Rapunzel syndrome.” This syndrome is characterized by the presence of a gastric foreign body, typically hair, which extends from the stomach into the small intestine. The overall incidence of trichobezoar is 0.5% globally [[2], [3], [4]].

It often occurs in young patients who suffer from trichotillomania (the compulsion to pull out one's hair) and trichophagy (the compulsion to swallow hair) [5]. Due to the stomach's high capacity, the mass may not cause symptoms until it grows too large. Once large enough, symptoms of obstruction may include recurrent abdominal pain, nausea, vomiting, anorexia, weight loss, and malabsorption of essential nutrients. If left undiagnosed, gastric bezoars can also lead to severe anemia, either due to malabsorption or gastrointestinal bleeding. Smaller gastric bezoars can often be removed minimally invasively, such as via endoscopy. However, larger trichobezoars, which are typically non-fragmentable, require open surgical intervention [6,7].

Trichotillomania (TTM), recognized in the DSM-5 as part of the obsessive-compulsive and related disorders spectrum, commonly coexists with comorbid conditions, particularly anxiety and depression, affecting around 80% of individuals with TTM [8,9]. This disorder can present as a symptom within various psychological conditions, including OCD, borderline personality disorder, schizophrenia, intellectual disability, and depression. In children, TTM can lead to trichobezoars (hairballs formed from hair-pulling and ingestion), often accompanied by intellectual disabilities, pica, or additional mental health challenges, which complicate diagnosis and treatment [10].

This case report discusses a trichobezoar classified as Rapunzel syndrome in a 19-year-old female in late adolescence and emphasizes the key aspects of diagnosis and treatment. It is propably the first case reported from this region.

## 2 Case report

A female in her late adolescence presented to the surgery OPD with continuous dull pain in her abdomen for past 6 months, recurrent episodes of vomiting since 4–5 years and chief complaints of lethargy and tiredness from past 8–10 years.

The patient was apparently asymptomatic until her menarche, thereafter her parents noticed growth retardation and low energy, unable to cope up with studies and sports in school in comparison to other students of her age, on and off complaint of non-itchy skin rashes which healed on its own, intermittent episode of vomiting containing food residues which was non bilious, recently she noticed lump which was not painful and thus it was brought into her parents notice and was shown to a doctor.

Demographics: The patient belonged to the rural community of Deoria district in Eastern Uttar Pradesh

*On gross examination,*

The patient weighed 31 kg and height of 142 cm with BP of 107/58 cm Hg and pulse rate 131/min at the time of 1st visit. The patient's height followed the IAP growth chart. The patient was also well oriented to time, place and person. The patient showed nutritional deficiencies such as scaly skin and spoon shaped nails (koilonychia), alongside peripheral tingling, accompanied by allied symptoms including urge to eat mud (pica), and mild anemia. Peripheral tingling suggests possible neuropathy due to nutritional deficits, likely caused by chronic pica or malabsorption.

Inspection: palpable abdominal lump in the upper abdomen, more prominent in the epigastrium and left hypochondriac region, extending towards the umbilicus. The lump was identified during the routine physical examination and was found to be mobile in all four directions. It was non-pulsatile and did not cause severe pain (non-tender). The examination revealed that we could palpate above the lump, suggesting it did not arise from retroperitoneal structures.

Palpation: can get above the swelling, non-tender, mobile in all direction,

Percussion: dull note.

Auscultation: no bruit. No succession splash.

Past history: The parents report a history of pica in the patient, stating that she used to eat chalk and hair when she was a toddler. However, the patient did not confirm or recall engaging in such behavior, as she does not remember it.

Other relevant history- The patient had normal menstrual history and made repeated visits to the doctor for her complaints in the past, though she primarily relied on home and Ayurvedic remedies for treatment. She was sexually inactive, denied any drug or physical abuse, and occasionally reported abdominal pain, which was managed with home remedies and medications from local shops.

*Investigations done:*

**Fig. 1:**
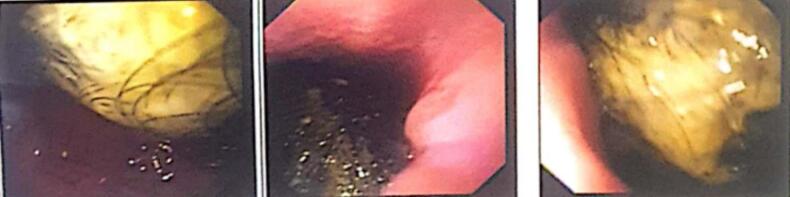
Upper gastrointestinal endoscopy showing large mass of hairball almost completely filling the stomach

**Fig. 2:**
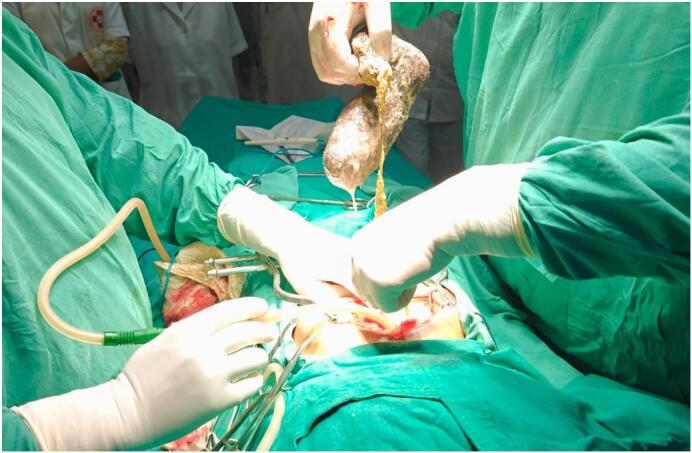
Intraoperative image of trichotellomania involving the entire stomach.

The blood investigation showed mild lymphocytosis and mild anemia. Abdominal ultrasound showed over distended stomach with slow gastric emptying (Fig. 1). To reach to a confirmatory diagnosis the patient was advised Upper G.I. endoscopy which was suggestive of Trichobezoar. The ECG and Xray chest findings were normal as preoperative tests. The patient was then planned for surgery for the lump excision (Fig. 2) and post operatively the patient was discharged and advised for routine followup.

Intraoperative findings: A midline laparotomy was performed from the xiphisternum to the umbilicus, and the abdomen was opened in layers. After breaching the peritoneum, the stomach was identified, and the surrounding organs were packed with a wet mop and retracted away from the operative field. A direct incision was made over the anterior surface of the stomach near the greater curvature, large enough to remove the entire specimen en masse. The abdominal cavity was thoroughly lavaged with normal saline. A Ryle's tube was inserted through the right nostril and advanced past the pylorus into the jejunum to establish a nasojejunal feeding route. The stomach wall was then closed in two layers using 2–0 round body silk sutures. The procedure was completed uneventfully. The patient was also advised cognitive behavioral therapy (CBT) with support from family for further improvement.

**Fig. 3:**
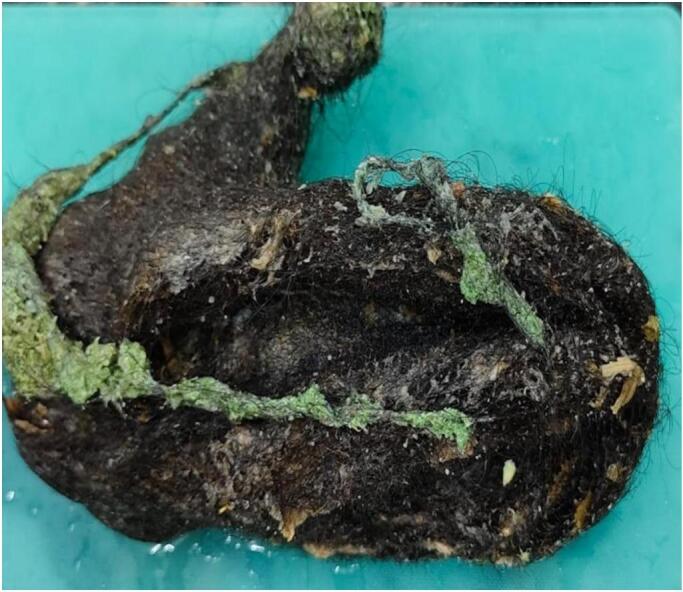
Fully excised gastric trichobezoar composed of hair and hair follicles extensions casted in the shape of stomach (20 x 13.5 x 9 cms).

Post operatively the mass was sent for biopsy for confirmatory diagnosis. On gross examination, the specimen composed of compact hair and hair extensions, extending from lower esophagus to pylorus measuring 20 x 13.5 x 9 cm and no other structure was grossly identified. On microscopic examination, hair follicles were only seen (Fig. 3).

*Differential diagnosis*

Differential diagnosis for the **palpable lump** includes:

**Gastric or pancreatic mass**: A tumor, cyst, or hypertrophic pyloric stenosis could explain the lump's position and the patient's vomiting history.

**Mesenteric mass**: Given the lump's mobility, a mesenteric cyst or lipoma is another consideration.

**Gastrointestinal disorders**: Chronic vomiting with a history of nutritional deficiency could suggest malabsorptive syndromes such as celiac disease or chronic gastritis.

## 3 Discussion

The case presented illustrates a novel clinical manifestation of Rapunzel syndrome and it is probably the first reported case in this region. The first documented case of a trichobezoar was reported in 1779 by Baudamant in a 16-year-old boy. The term “Rapunzel” originates from the fairy tale in the Brothers Grimm collection, in which the heroine is known for her extremely long, blonde hair. The first case of Rapunzel syndrome was published by Vaughan and colleagues in 1968. A total of 49 case reports of Rapunzel syndrome have been reviewed, with the age range of affected individuals spanning from 4 to 19 years [11].

The majority of patients have a psychiatric history of conditions such as trichotillomania, trichophagia, or pica (the consumption of non-nutritive substances like ice), often accompanied by behavioral changes. It is estimated that 1 in 2000 children suffer from trichotillomania, but it is severely underdiagnosed [12]. However, this history is very difficult to elicit upon initial presentation unless it is specifically asked for [13].

This condition typically remains asymptomatic for an extended period, with symptoms emerging only in the later stages. The symptoms are often nonspecific, including abdominal pain (46.66%), nausea and vomiting (44.44%), obstruction (20%), abdominal distension (8.88%), and weight loss (8.88%). The clinical and radiological features help us narrow the diagnosis [14].

The case highlights the importance of considering behavioral factors like pica in patients with gastrointestinal and nutritional symptoms. Chronic pica, particularly when untreated, can lead to serious nutritional deficits and gastrointestinal issues, including the formation of masses. Nutritional repletion and treatment of the underlying cause (potentially gastrointestinal or behavioral) will be key in managing this case. Furthermore, the upper G.I Endoscopy was suggestive of the disease.

In our case, the patient underwent exploratory laprotomy for the treatment which is similar to other case report from USA, Israel and Norway [15,16].

The patient's psychiatric course was closely followed in the hospital and post-operatively in the outpatient setting. Pharmacologic treatment with drugs like quetiapine may help control the obsessive behavior exhibited by patients who ingest hair extensions [17].

The patient chose to eat hair extensions because it helped temper her anxiety. She denied skin picking, pulling out her own hair while Jones et al. reported a case of Rapunzel syndrome where habit-reversal training, combined with behavioral therapy in a supportive family setting, played a key role in the post-operative period [17]. Additional therapeutic approaches, such as cognitive therapy and acceptance and commitment therapy, can further enhance the effectiveness of behavioral therapy in managing this challenging condition [18]. This case has been reported in line with the SCARE criteria [19].

## 4 Conclusion

This patient presents a complex interplay between gastrointestinal symptoms (including a palpable lump and vomiting), nutritional deficiencies (manifested as skin changes, koilonychia, and tingling), and a longstanding history of pica. Further diagnostic imaging and laboratory workup will help clarify the origin of the lump and the extent of the nutritional issues. Treatment will likely involve addressing both the physical mass and the underlying nutritional deficiencies.

## Written consent

Written informed consent was obtained from the patient for publication of this case report and accompanying images. A copy of the written consent is available for review by the Editor-in-Chief of this journal on request.

## Ethical approval

Not applicable.

## Guarantor

Dr Krati Agarwal

Senior Resident

AIIMS, Gorakhpur

9926967942

Email id- krati0192@gmail.com

## Research registration number

N/A.

## Sources of funding

Nil.

## Declaration of competing interest

Nil.
